# Is there really a pot of gold at the end of the rainbow? Has the Occupational Specific Dispensation, as a mechanism to attract and retain health workers in South Africa, leveled the playing field?

**DOI:** 10.1186/1471-2458-12-613

**Published:** 2012-08-06

**Authors:** Gavin George, Bruce Rhodes

**Affiliations:** 1 Health Economics and HIV and AIDS Division, University of KwaZulu-Natal, Durban, South Africa; 2 School of Accounting, Economics and Finance, University of KwaZulu-Natal, Durban, South Africa

**Keywords:** Human Resources for Health (HRH), Salaries, Occupation Specific Dispensation (OSD), Migration, Purchasing Power Parity (PPP)

## Abstract

**Background:**

South Africa is experiencing a critical shortage of human resources for health (HRH) at a time when the population and the burden of ill-health, primarily due to HIV, AIDS and TB, are on the increase. This shortage is particularly severe within the nursing profession, which has witnessed significant emigration due to poor domestic working conditions and remuneration. Salaries and other benefits are an obvious pull factor towards foreign countries, given the often extreme international wage differentials. The introduction of the Occupation Specific Dispensation (OSD) in 2007 sought to improve the public services’ ability to attract and retain employees thereby reducing incentives to emigrate.

**Methods:**

Using a representative basket of commonly bought goods (including food, entertainment, fuel and utilities), a purchasing power parity (PPP) ratio is an exchange rate between two currencies that equalises the international price of buying that basket. Our study makes comparisons, using such a PPP index, and allows the identification of real differences in salaries for our selected countries (South Africa, United States, United Kingdom, Canada, Australia and Saudi Arabia) for the same HRH professions. If PPP adjusted earnings are indeed different then this indicates an economic incentive to emigrate.

**Results:**

Salaries of most South African HRH, particularly registered nurses, are dwarfed by their international counterparts (notably United States, Canada and Saudi Arabia), although the OSD has gone some way to reduce that disparity. All selected foreign countries generally offer higher salaries on a PPP adjusted basis. The United Kingdom ($43202) and Australia ($38622), in the category of Medical Officer, are the only two examples where the PPP adjustment brings the salary below what is being offered in South Africa ($50013 post OSD). The PPP adjusted salary differences between registered nurses is very slight for South Africa ($18884 post OSD), Australia ($21784) and the United Kingdom ($20487). All other foreign countries show large salary advantages across the HRH categories examined.

**Conclusion:**

Whilst South African salaries remain lower than their foreign counterparts by and large, the introduction and implementation of the OSD has made significant progress in reducing the gap between salaries of HRH in South Africa (SA) and the rest of the world. Given that the OSD has narrowed the gap between SA and overseas salaries whilst in the context of continued out migration of SA HRH, further research into push factors effecting migration needs to be undertaken.

## Background

According to a factsheet produced by the World Health Organization (WHO) [1], by 2006 there were 59.8 million health workers globally involved in the provision of health services or the management and support thereof. These individuals are commonly referred to as human resources for health (HRH); the individuals whose job it is to protect and improve the health of their communities. Fifty-seven countries worldwide have been identified as having a critical shortage of HRH, the majority located in Africa and Asia. This global shortage collectively amounts to 4.2 million HRH, with medical officers, nurses, and midwives accounting for approximately 2.4 million of this number [1]. Reasons for this shortage vary between regions, with common factors being retirement, migration, death, low production rates, and poor working conditions [1].

South Africa’s medical officer and nurse density ratios are favourable compared to other southern African countries. Based on its density of medical practitioners to population ratio, South Africa is ranked slightly higher than low income countries which have a ratio of 50:100 000, but is hugely under-resourced in comparison to other middle and high income countries that have ratios of 180:100 000 and 280:100 000 respectively [2]. Estimates for 2010 have put the number of nurses and doctors (medical officer and specialists) working in South Africa at 221 817 (2009 data), 17 801 and 9 630 respectively, which implies a nurse and doctor to patient ratio of 410 and 55 per 100 000 population [3]. South Africa’s medical officer to population ratio falls way below countries with a similar level of economic development such as Mexico (198 per 100 000) and Brazil (185 per 100 000) [3]. Conversely, the nursing population ratio is more favorable than Mexico (400 per 100 000) and Brazil (290 per 100 000) [4]. These data indicate that South Africa’s combined medical practitioner and nurse density ratios are above the minimum level of 230:100 000 recommended by the WHO [5]. However, these favorable density ratios hide internal disparities particularly between provinces and between the public and private health sector, as well as urban and rural areas.

The migration of skilled public sector employees led to the development and implementation of the Occupation Specific Dispensation (OSD) in 2007 by the South African government. The OSD aimed at improving the conditions of service and remuneration for public service workers, including public sector health professionals. The objectives of the OSD were to improve the public services’ ability to attract and retain employees, to provide differentiated remuneration dispensations for the vast number of occupations in the public service, to cater for the unique needs of the different occupations, to provide a unique salary structure per occupation, to prescribe grading structures and job profiles to eliminate inter-provincial variations and to provide adequate and clear salary progression and career path opportunities based on competencies, experience, and performance [6,7].

Whilst there are a number of reasons for the attrition of HRH, this article will focus on the overseas migration of HRH influenced by salaries and benefits as a pull factor.

### HRH migration in South Africa

In 2008, there were roughly 250 000 HRH employed in South Africa’s health system, similar to the number in 1997/98. After taking into account population growth and the burden of disease, the Development Bank of South Africa calculated a staff shortage of 79 791 HRH in 2007/08 [8]. This critical shortage of HRH is being experienced at a time when the population and the burden of ill-health, primarily due to HIV, AIDS and TB, are increasing among the population [9]. Africa Health Placements, an NGO that recruits doctors to work in under-serviced rural areas in South Africa, estimated that half of the 2 400 South African medical graduates in 2006 and 2007 would leave the country; of the remaining 1 200 medical officers, 75% would work in the private sector, leaving 300 to work in the public sector; of those 300, possibly 70 would work in the public health services in a rural facility [10].

Whilst there is no agency that collects standardised data on international migration flows disaggregated by occupation, research estimated the amount of African-born doctors working abroad using census data [11]. This data suggests that in 2001 about 25% of all South African-born doctors were working in seven other countries. For that same year they also estimated that roughly 5% of South African professional nurses worked abroad, which is equivalent to approximately 4844 in 2001. This study reaffirms an earlier study which reports that since 1975, 45% of University of Witwatersrand medical school graduates were located abroad, mostly in North America, the United Kingdom, Canada, Australia and New Zealand [2]. In the mid-2000s, there were reportedly more than 20 overseas commercial recruitment agencies working locally to recruit South African qualified doctors, with 10% of Canada’s hospital-based physicians having graduated from South African medical schools, and 6% of the United Kingdom’s total health workforce, including professionals (nurses and doctors), trained in South Africa [10].

The Southern African Migration Project (SAMP) reported in 2008 that half of South Africa's health professionals planned to emigrate within the following five years [12].

### Reasons for the attrition of HRH in South Africa

This attrition of HRH is attributed to push and pull factors depending on whether the factor is located in the source or destination country. Prominent push factors for HRH in sub-Saharan Africa include resource limited health care systems, deteriorating work environments, human resource shortages, low salaries, political tensions, gender discrimination, lack of personal security, HIV/AIDS, and deteriorating quality of life and social systems such as education and welfare [11,13-16]. Salient pull factors include the availability of jobs in the destination country, more manageable workloads, high remuneration, better working conditions, safer living environments, better quality of life and a more economically and politically stable country [11,13-16]. A South African study identified financial factors, better job opportunities, schooling opportunities for children abroad and the high crime rate in South Africa, as significant factors encouraging emigration [17].

Table 1 illustrates salary differentials across selected HRH categories in selected destination countries. The South African data displays pre- and post-OSD salaries for each HRH category. As we can see, the new OSD salary structure has raised South African salaries by over half in some cases.

**Table 1:** Selected gross HRH salaries in national currency units and US dollars

**NCU data**	**SA (Rand)**^**a**^		**UK (£)**	**AUS ($)**	**CAN ($)**	**US ($)**	**Saudi Arabia (SR)**
	**Pre OSD**	**Post OSD**^**b**^					
Prof Nurse	1105220 ($12416)^^^	160032 ($18884)	23019^3^ ($34758)	55354^6^ ($41294)	63004^8^ ($53679)	62000^11^	199176^13^ ($53180)
Med Officer Grade 2	247512 ($29206)	423846 ($50013)	38000^4^ ($57380)	98138^7^ ($73211)	121687^9^ ($103677)	181000^12^	295200^14^ ($78818)
Specialist Grade 2	461757 ($54487)	554109 ($65385)	95748^5^ ($144580)	325000^7^ ($242450)	320116^10^ ($272739)	321000^12^	440160^14^ ($117523)

^Figures in parentheses show average 2009 US dollar market rate equivalent [18].

^1^[19]^2^[20]^3^[21]^4^[22] average of range. ^5^[23] average of range ^6^[24]^7^[25] average of range ^8^[26]^9^[27]^10^[28]^11^[29]^12^[30]^13^[31]^14^[32].

^a^The SA data do not contain a rural allowance. Health workers operating in rural areas get an extra 18%.

^b^The OSD increase for South African nurses came into effect on 1^st^ July 2008 and represents a 24% increase for the registered nurse [9].

Despite the introduction of the OSD, all selected foreign countries offer salaries that are considerably higher than their South African counterparts. This paper sets out to use these data as a baseline from which to make a truer comparison, namely using PPP ratios.

### The Occupation Specific Dispensation (OSD) for health professionals

The development and implementation of the OSD arose from the recognition that improvement in the conditions of service and remuneration for health professionals constituted an urgent priority. Announced in 2007, the OSD resulted in all HRH in the public service being re-graded according to their qualifications and years of experience with their remuneration increasing [33]. It was agreed that nursing would be the first profession to benefit from the OSD.

With regard to nurses, it was agreed that there would be two phases to the salary adjustment, followed by a minimum adjustment to nurse salaries in line with the OSD and, secondly, there would be a recalculation and progression based on recognition of relevant experience. As part of phase 1, entry level salaries for professional nurses and nursing assistants were increased by 24%, while entry level salaries for enrolled nurses were increased by 20% [34].

The entry level OSD salary in July 2007 for nurses prior to phase 2 of the OSD, when relevant years of experience were taken into account, was R106 086 ($12525) for professional nurses, R70 140 ($8281) for enrolled nurses, and R53757 ($6347) for nursing assistants. The salary notches of nurses were further increased in July 2008 and again in July 2009. These appear in Table 2. The entry level salary notches of professional nurses employed in general nursing positions increased by 10.5% for each grade from 2007 to 2008, followed by an increase of between 10.5% and 11% in 2009. In comparison to the entry level salary of professional nurses of R85 362 ($10078) prior to the OSD, presented in Table 2, the entry level salary notch for professional nurses (Grade 1) in July 2009 reflects a 52.4% increase.

**Table 2:** Remuneration for professional nurses, pre- and post changes to the OSD, 2007-2009

**Service/ experience**	**Revised production grade**	**Annual salary notches as at 1 July 2007**	**Annual salary notches as at 1 July 2008**	**Total annual salary packages as at 1 July 2008**	Annual salary notches as at 1 July 2009*	**% increase in salary 07-08**	**% increase in salary 08-09**
< 4 years	Grade 1	R106 086 ($12525)^^^	R117 225 ($13840)	R174 333 ($20582)	R130 119 ($15362)	10.5%	11%
5–9 years	Grade 1	R115 923 ($13686)	R124 365 ($14683)	R182 996 ($21605)	**		
10–19 years	Grade 2	R130 473 ($15404)	R144 174 ($17022)	R207 031 ($24443)	R160 032 ($18894)	10.5%	11%
20–29 years	Grade 3	R160 470 ($18946)	R177 318 ($20935)	**	R195 936 ($23133)	10.5%	10.5%
> 30 years	Grade 3	R186 030 ($21963)	R205 563 ($24270)	R281 516 ($33237)	**		

^ figures in parentheses show average 2009 US dollar equivalent [18].

*OSD for nursing personnel taking into account cost of living adjustment [19].

** No data available.

Medical, dental, specialists, pharmacists, and emergency medical services (EMS) were identified for implementation in 2008. However, due to inadequate funding, salary adjustments could only be implemented in 2008/2009 [6]. Medical interns experienced a substantial increase of between 31% and 53% in their salary package. Categories of medical professionals, such as community service medical officers, medical officers in Grade 1, 2, and 3 saw significant salary adjustments of up to 68% with medical specialists in Grade 1, 2, and 3 posting increases of between 2 and 50% in Year 1 of implementation. This was followed by further significant increases across medical categories in Year 2, as illustrated in Table 3[6].

**Table 3:** Remuneration for medical officers and specialists with percentage increases at Year 1 and Year 2

**Professional category**	**Grade**	**Experience/ service**	**Annual salary prior to OSD**	**Annual salary as at 1 July 2009**	Total annual salary package as at 1 July 2009*	**% Increase Year 1**	**% Accumulative increase Year 2**
Medical intern			R117 501 ($13873)^^^	R228 828 ($27016)	R314 023 ($37075)	31 % - 53%	
Medical officer (Community service)			R174 243 ($20572)	R286 086 ($33776)	R392 599 ($46352)	9.8 % - 18.9%	6.1%
Medical officer	Grade 1	0 – 4 years	R174 243 ($20572)	R332 016 ($39199)	R365 217 ($43119)	9 % - 25%	12%
	Grade 2	5 – 9 years	R247 512 ($29222)	R385 314 ($45492)	R423 846 ($50041)	26 % - 44%	14%
	Grade 3	> 10 years	R391 026 ($46166)	R447 174 ($52795)	R491 892 ($58075)	46 % - 68%	20%
Medical specialist	Grade 1	0 – 4 years			R491 892 ($58075)	1.8 % - 18%	13%
	Grade 2	5 – 9 years			R554 109 ($65420)	14.7 % - 32.9%	20%
	Grade 3	> 10 years			R624 198 ($73695)	29.2 % - 49.8%	26%
Principal specialist			R769 271 - R962 174 ($90823-113598)		R932 399 ($110083)	4.6 % - 25.1%	
Chief specialist			R932 399 ($110083)		R1.2 million ($141677)	7.9 % - 29%	

^ figures in parentheses show average 2007 US dollar equivalent [18].

*Figures represent the OSD remuneration packages after cost of living adjustments as at 1 July 2009. Source: [20]; [6].

Given the new South African OSD dispensation described above, this paper examines how that new salary structure compares to selected foreign countries in comparable HRH categories using PPP adjustments. Country choice was based on historical migration patterns where South African HRH migrants tend to target countries such as the United Kingdom, Australia, Canada and the United States [2]. Saudi Arabia is included as a representative Gulf state due to the increasing flow of HRH to the Gulf region. It is estimated that 80 % of HRH, in the Gulf, are foreign trained [35]. Whilst Saudi Arabia is well known for its tax-free status, it also provides additional benefits such as free (furnished) accommodation, medical care (especially for those working in a hospital), emergency dentistry support and extensive leave are common place [31]. The earnings available in Saudi Arabia make these additional benefits trivial in terms of understanding HRH migration patterns.

## Methods

The existing literature examines actual salaries across health professions offered in different, popular destination countries [2]. For more informed comparisons on earnings, we require a purchasing power parity (PPP) index. Using a representative basket of commonly bought goods (including food, entertainment, fuel, and utilities), the PPP is an exchange rate between two currencies that equalises the international price of buying that basket [36].

Our study makes comparisons using this PPP index approach that has been used in other health salary studies for sub-Saharan countries [37]. Foreign salaries expressed in national currency units were converted to the Rand equivalent using PPP ratios published by the International Monetary Fund (IMF) [38]. The PPP is essentially an exchange rate between two countries that equalises the cost of living. It is founded on the principle of an international dollar. This is a purely hypothetical currency where one international dollar (earned in another country) has the same purchasing power as one US dollar earned and spent in the US. The PPP for the United States is set at 1.0 and acts as a baseline to compare all other countries. Examining the cost of living adjusted salaries for each of our selected countries allows the identification of financial incentives to emigrate within a given HRH profession.

The PPP figures are presented relative to South Africa and are shown in Table 4. These figures are labeled Rand-based PPP ratio because they are the exchange rates that allow the comparison of all purchasing power differences relative to South Africa (not the United States). PPP data is typically expressed relative to the United States dollar. For instance, if the United Kingdom has a PPP of 0.65 then this is the PPP exchange rate between itself and the United States only. To compare the purchasing power of South Africa relative to the United Kingdom, we need to take the UK£-US$ PPP (0.65) and the South African Rand-US$ PPP (4.9) to generate the South African Rand-UK£ PPP (4.9/0.65) of 7.53. Thus R75300 ($8890) earned and spent in South Africa has approximately the same purchasing power as £10000 ($15100) earned and spent in the United Kingdom.

**Table 4:** HRH gross salaries by national currency unit, Rand-foreign currency market exchange rate and PPP (Rand-based) equivalent

	**SA (Rand)**^**a**^		**UK (£)**	**AUS ($)**	**CAN ($)**	**US ($)**	**Saudi Arabia (SR)**
	**Pre OSD**	**Post OSD**^**b**^					
**Registered Nurse**	R105220^1^	R160032^1^	23019^3^	55354^6^	63004^8^	62000^9^	199176^14^
	($12416)^	($18884)	($34758) [R302700]^c^	($41294) [R360908]	($53679) [R463079]	[R525140]	($53180) [R450138]
Market and (PPP)^e^ foreign exchange rates^d^			13.15 (7.54)^e^	6.52 (3.33)	7.35 (4.08)	8.47 (4.9)	2.26 (2.06)
PPP salary (Rand-based) ^d^			R173528	R184513	R257266	R303800	R410068
			($20487)	($21784)	($30374)	($35868)	($48414)
**Med Officer**	R247512^2^	R423846^2^	38000^4^	98138^7^	121687^10^	181000^13^	295200^14^
**Grade 2**	($29206)	($50013)	($57380) [R638314]	($73211) [R639860]	($103677) [R1163387]	[R1533070]	($78818) [R667152
		PPP salary (Rand-based)			R365925^c^	R327127	R646326	R886900	R607764
			($43202)^f^	($38622)	($76308)	($104711)	($71755)	
**Specialist**	R461757^2^	R554109^2^	95748^5^	325000^7^	320116^11^	321000^13^	440160^14^	
**Grade 2**	($54487)	($65385)	($144580) [R1158551]	($242450) [R221975]	($272739) [R2304835]	[R2375400]	($117523) [R867115]	
PPP salary (Rand-based)			R721793^c^	R1083333	R1307140	R1572900	R906211	
			($85218)	($127902)	($154326)	($185702)	($106991)	

^ figures in parentheses show average 2009 US dollar equivalent [18].

^1^[19]^2^[20]^3^[21]^4^[22] average of range. ^5^[23] average of range. ^6^[24]^7^[25] average of range ^8^[26]^9^[27]^10^[28]^11^[29]^12^[30]^13^[31]^14^[32].

^a^ The SA data does not contain a rural allowance. Health workers operating in rural areas get an extra 18%.

^b^ The OSD increase for South African nurses came into effect on 1^st^ July 2008 and represents a 24% increase for the registered nurse [9].

^c^Rand equivalent of foreign salary in square parentheses (2009 monthly average market exchange rates[18]).

^d^Market exchange rates in this row are used for all Rand calculations.

^e^Author calculations. Figures in parentheses show PPP exchange rates between Rand and foreign currency.

^f^Author calculations. Figures in parentheses show US$ equivalent (using market rate) of PPP Rand-based salary.

It is worth noting that the PPP approach is not without its problems. It can be difficult to find a comparable basket of goods for all countries. Availability and quality of goods are only two issues which hinder the establishment of valid basket comparisons. Other relevant factors are tax rates, however, our PPP calculations and trends between country earnings vary little between gross and net comparisons and only gross earnings comparisons have been made.

### Selected employment categories and countries

Three categories of state-employed HRH - a registered or professional nurse with 3 to 5 years’ experience, a medical officer (level 10, Grade 2) and a specialist doctor (consultant, level 12, Grade 2) - were chosen for the international salary comparisons within the respective country’s public sector.

### Health worker definitions

Across the five countries a South African professional nurse is equivalent to a foreign based registered nurse. The defining point for this category is that the worker has a formally recognised nursing degree. A Grade 2 medical officer requires a medical degree to work in a general hospital having 5 to 9 years’ experience. The chosen experienced position is a medical specialist (grade 2 also of 5 to 9 years’ experience). These terms and definitions are easily recognised internationally and this affords more accurate comparisons.

### Data

Various sources were found for each of the six target countries to obtain salary data for the above HRH positions over the 2008/9 period. For a given HRH position and country, the clearest and most detailed source was selected for the comparison in Table 4. Some discrepancies between sources are inevitable but figures identified were felt to be the most representative. When only ranges were available, medians or averages were calculated.

## Results and discussion

South African data is generally treated as the baseline showing Rand salaries before and after OSD implementation. The Rand salaries are then converted to the foreign country equivalent using market exchange rates and finally converted to PPP equivalents. For instance, in Table 4, a nurse earning £23019 ($34758) in the UK will be earning the equivalent of R302700 (in square brackets) if the market exchange rate were R13.15 to the British pound. Alternatively, using a PPP ratio of R7.54 to the British pound, the Rand equivalent falls to R173528 ($20487) since the PPP exchange ratio captures the differences in the cost of living between the UK and South Africa.

These sets of comparisons, particularly the PPP adjusted figures, give a useful insight into how the post OSD South African HRH salary structure may influence decisions to emigrate based on remuneration.

### Salary comparisons

The salary comparisons using national currency units and PPP adjusted conversions are presented in Table 4. We begin with registered nurses showing the salaries across the chosen countries. The following row then shows the market exchange rate of Rand to foreign currency. That currency is then used to calculate the Rand equivalent of a foreign-based worker. The same calculations are applied to grade 2 medical officers and specialists using the same exchange and PPP rates as for registered nurses. As we can see from Table 4, when an HRH worker earning Rands in South Africa performs a simple, market-based exchange rate calculation converting a foreign salary to Rands, the results show that foreign-based HRH are in a much better position and hence this may encourage South African HRH to migrate. Of course the comparison is not valid as the market exchange rate does not capture differences in living expenses which can be considerably higher in foreign countries. The PPP recalibrates the country comparisons and whilst brings foreign earnings closer to South African salaries, the gaps are still significant.

Table 4 shows that all countries offer higher salaries on a PPP adjusted basis. The United Kingdom and Australia, in the category of medical officer are the only two examples where the PPP adjustment brings the salary below what is being offered in South Africa. The PPP adjusted salary differences between registered nurses is very slight for South Africa, Australia and the United Kingdom. All other foreign countries show large salary advantages across HRH categories examined.

At this point it is worth noting the phenomenon of ‘wage-disadvantage’ when comparing native with migrant workers. This is not necessarily a phenomenon pertinent to our selected foreign countries or even for HRH, but nonetheless as a general international labour market effect, some evidence has been found to suggest that native workers are offered higher salaries compared to equally qualified migrant workers, especially from non-European countries [39,40]. If this is the case, then incentives to emigrate, identified above, will be reduced. Whilst there is evidence to suggest that some ‘wage-disadvantage’ in this context is a real phenomenon, it is not considered significant here and no further detail is deemed necessary since the wage differentials identified are very large.

It is clear that the salaries of most South African HRH are dwarfed by their international counterparts, although the OSD has gone some way to reduce that disparity. It is interesting to note that the improvement of salaries of South African medical officers has resulted in net earnings surpassing their counterparts in the United Kingdom and Australia. This discordant data, however, lends support to research which suggests that push factors (mainly working conditions) outweigh pull factors such as increases in remuneration gained abroad in health worker’s emigration decisions [11]. This research finds that HRH in the public and private sectors in South Africa report that job dissatisfaction, delayed salaries, delayed promotions and lack of recognition in the workplace are central to their reasons for wanting to emigrate.

The introduction and implementation of the OSD has made significant progress in reducing the wage gap between HRH in South Africa and the rest of the world. Of concern, is the fact that this gap is unlikely to close any further. The South African Minister of Finance, in his 2010 budget speech, stated that “the 2009 round of salary increases has placed immense pressure on the budget. Including the very necessary adjustment to the salaries of professionals, the wage bill has almost doubled in five years. Now that a major revision to public service remuneration is behind us, it will be necessary to moderate salary increases going forward.” This statement suggests that South African health care workers should only expect moderate salary increases for the foreseeable future [41].

As much as stronger foreign currencies lure South African HRH to migrate, it should be recognized that there are HRH operating in poorer countries who are in turn enticed by the salaries offered within South Africa. Over the years, South Africa has benefitted from the recruitment of foreign doctors from elsewhere in Africa and Cuba, some of whom were recruited by the Department of Health to work in under-served areas [15].

It is important to recognize that the migration of HRH to other countries is less than the numbers who move from the public to the private sector [7]. The introduction of the OSD was implemented to slow or try to halt this movement and encourage HRH to move back into the public sector. There are currently insufficient data to determine if the new salaries brought about through the introduction of the OSD has resulted in a slowdown of this public to private movement.

It should also be noted that South African public sector HRH may yet be enticed into the private sector within the developed countries selected for review in this study. Unfortunately insufficient wage data is available for the private sector both within South Africa and the comparison countries.

## Conclusion

This article highlights the financial improvement the OSD has made to the salaries of South African HRH. More interesting is that the post OSD South African medical officer salary ($50013) surpassed foreign based counterparts by 7 to $12000 a year for the United Kingdom ($43202) and Australia ($38622) respectively under PPP adjustment. In addition, the OSD has gone some way to rectifying the international salary imbalances which remain within the South African private and public health care sector. It is this discordant data which highlights that HRH are not migrating for better salaries alone, but rather, there are a number of push factors which fuel migration.

Health worker migration is a problem faced by a number of lower middle income countries (LMIC) and whilst an increase in salaries in these countries may have an effect on HRH movements it shouldn’t be the only factor countries seek to address. Factors relating to the working conditions of health professionals in the public service remain salient considerations in health professionals’ reasons for leaving the country, and potentially carry more weight in their decisions than the pull factors that First World countries can provide. Such factors require future investigation.

The OSD has been implemented by the South African government in an attempt to alleviate the HRH crisis and strengthen efforts to attract and retain HRH to the public health sector. Whilst the improved remuneration package of all categories of health professionals is to be lauded, it is imperative that working conditions are further improved within the public health sector to maintain this momentum.

## Competing interests

The authors declare there are no competing interests.

## Authors’ contributions

GG conceptualized the study and prepared the initial draft. BR conducted the analysis and contributed to writing the manuscript. Both authors read and approved the final manuscript.

## Pre-publication history

The pre-publication history for this paper can be accessed here:

http://www.biomedcentral.com/1471-2458/12/613/prepub
